# A family caregiver’s relaxation enhances the gastric motility function of the patient: a crossover study

**DOI:** 10.1186/s13030-015-0048-y

**Published:** 2015-10-31

**Authors:** Hideaki Hasuo, Kenji Kanbara, Yasuyuki Mizuno, Junji Nishiyama, Mikihiko Fukunaga, Naoko Yunoki

**Affiliations:** Department of Psychosomatic Medicine, Kansai Medical University, Shinmachi 2-5-1, Hirakata, Osaka 573-1090 Japan; Akaiwa Medical Association Hospital, Akaiwa, Japan

**Keywords:** Family caregiver, Self-care, Sense of guilt, Relaxation, Gastric motility function

## Abstract

**Background:**

The primary purpose of this study was to assess the effect of a caregiver’s relaxation on the gastric motility function of the patient. The secondary purpose was to evaluate changes in the caregiver’s willingness to perform self-care following feedback on the results of the primary purpose.

**Methods:**

Subjects were 26 patients with a decreased level of consciousness who received gastrostomy tube feeding and their 26 family caregivers. We compared the patient’s gastric motility under the condition of having his or her hand held with and without caregiver relaxation (crossover study). Changes in the caregiver’s willingness to perform self-care following feedback on the results was evaluated using self-administered questionnaires. Hypnosis was used for relaxation. The outcomes assessed for gastric motility function were the motility index and gastric emptying rate by ultrasonography examination.

**Results:**

Hand-holding by the family caregiver while he or she was receiving relaxation enhanced the patient’s gastric motility function. By giving feedback on the results, the caregiver’s willingness to adopt self-care was increased and his or her sense of guilt was reduced.

**Conclusions:**

This study suggested that a caregiver’s relaxation increases the gastric motility function of the patient and that gettinng feedback including the positive results increases the caregiver’s willingness to perform self-care, which consequently reduce the caregiver burden.

## Background

Caregiver burden is defined as “a degree of emotional, physical, social and financial stress resulting from taking care of the family member,” and it is important to take an effective approach to reducing caregiver burden [1]. Self-care is important for reducing the family caregiver’s burden. Many families of a patient with dementia experience a decrease in their sense of happiness. This decrease in happiness is especially intense when the caregiver’s valued activities and interests are extremely limited [2].

In terms of the caregiver’s self-care, a report has suggested the usefulness of relaxation techniques for the family members of a dementia patient [3]. However, many caregivers cannot spare the time to engage in self-care because they experience a sense of helplessness regarding their relationship with the patient and also feel guilty for performing self-care activities [4]. It has been reported that it is common for family members of cancer patients not to have their psychological needs satisfied, such as overcoming a sense of guilt [5].

To our knowledge, no study has evaluated an intervention to increase caregiver willingness to adopt self-care, such as relaxation. One study suggested that a sense of guilt is reduced when the family interacts meaningfully with the patient [6]. Thus, we hypothesized that sharing the evaluation of the influence of the caregiver’s relaxation on the patient’s autonomic nervous function might increase the caregiver’s willingness to adopt self-care, such as relaxation and could eventually reduce caregiver burden.

In a previous study, we objectively evaluated the influence of hand-holding on the patient’s gastric motility function using gastrostomy tube feeding and ultrasonography [7]. We founded that the patients’ motility index (MI) and gastric emptying rate (GER) were significantly increased under hand-holding conditions. The family caregiver’s self-efficacy was also improved by explaining the positive results. Furthermore, a relationship between autonomic nervous function and gastrointestinal motility function has long been reported [8]. Although gastric motility function has been measured with internal pressure or radio isotope methods, those methods are difficult to use in everyday clinical practice because they are invasive. The gastric motility test using ultrasonography is not yet widespread, but its clinical efficacy has been supported in previous studies [9, 10]. We selected this method because it is simple, less invasive and capable of evaluating the function objectively and with high reproducibility because of the quantitative assessment of multiple gastric motility functions.

Hypnosis artificially induces a state characterized by increased suggestibility and altered behavior, perception, memory and thought (an altered state of consciousness) [11]. Because hypnosis can be used to induce relaxation, we evaluated the association between hypnosis and gastric motility function using ultrasonography [12], finding that hypnosis affected the autonomic nervous system and significantly accelerated antral gastric motility and proximal gastric distension in healthy people.

The primary purpose of the present study was to assess the effect of the caregiver’s relaxation induced by hypnosis on the patient’s gastric motility function, assessed by ultrasonography examination. The secondary purpose was to evaluate the change in the caregiver’s willingness to perform self-care following feedback on the results of the primary purpose, using a self-administered questionnaires.

## Methods

### Participants

In total, 26 patients with a decreased level of consciousness who receive gastrostomy tube feeding and their 26 family caregivers participated in this study. We explained the purpose and the methods of the study, the possibility of withdrawal from the study, and the protection of personal information to the family members of all study participants. We conducted the study with only those family caregivers who gave the informed consent. This study was conducted with the approval of the institutional board of Akaiwa Medical Association Hospital.

Of the 26 patients, 13 were male and 13 female. The average age was 78.3 years old (SD = 9.2). The degree of consciousness disorders was an irreversible state in which communication could hardly take place (Glasgow Coma Scale: 3–7 points, where 3 indicates deep unconsciousness and 15 indicates normal consciousness). The patients had been hospitalized for care purposes, and their physical conditions were relatively stable, with gastrostomy performed in the past. Cases with a high degree of esophageal hiatal hernia were excluded.

Of the 26 family caregivers, four were male and 22 female, with an average age of 61.3 years old (SD = 10.2). The relationships with the patients included two husbands, nine wives, five daughters, three grandchildren, four siblings and three other types of relatives.

### Study design

**Fig. 1:**
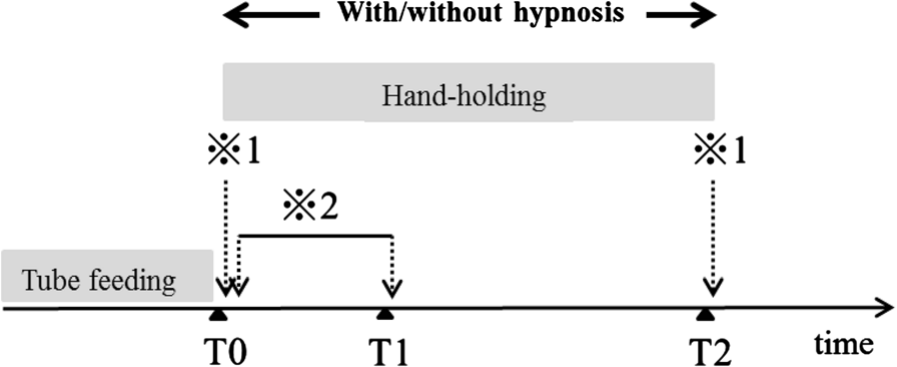
The assessment of the patient’s gastric motility under the condition of having his or her hand held with and without caregiver relaxation. T0: the end of tube feeding. T1: 3-min after the end of tube feeding. T2: 60-min after the end of tube feeding. 1: measure if the antral cross-sectional area. 2: measure of the number of contractions and the contraction rate for 3-min period after the end of tube feeding

We assessed the patient’s gastric motility under the condition of having his or her hand held with and without caregiver relaxation, and compared the results (crossover study) (Fig. 1).

Hypnosis was used for relaxation. This study was performed in a single session, and a clinically applicable technique was not used. The first author conducted the hypnosis, progressing through the stages of introduction, induction and deepening while evaluating the participant’s response. The participant was invited to go deeper into relaxation in response to the metaphor of a countdown from 10 to 0. A permissive approach was used throughout most of the trance session. The participants were invited to imagine a relaxed and pleasant day on holiday. The desired hypnotic state was hypnoid-light hypnosis, and this was assessed by measuring the depth based on the emergence of muscle relaxation and catalepsy.

The outcomes for gastric motility function were the MI and GER of the ultrasonography examination. MI was calculated by multiplying the number of contractions by the contraction rate for the 3-min period after the end of tube feeding. The contraction rate was defined as the average rate of changes in the antral cross-sectional area between the contraction phase and the relaxation phase. GER was assessed as the rate of changes in the antral cross-sectional area at the relaxation phase between the end of tube feeding and 60 min thereafter. The evaluation procedure was as follows:

1. Discontinue the administration of nutritional agents on the morning of the test day.
2. Prepare the test meal (thick liquid diet 300 kcal/200 ml + warm water 200 ml: 300 kcal/400 ml).
3. In Fowler’s position of 30°, at noon start administration of the test meal via gastrostomy at a rate of 200 ml/h.
4. Over time, measure the antral cross-sectional area and the number of contractions using ultrasonography. The observations were performed for the 3-min period after the end of tube feeding and at 60 min after the end of feeding. To visualize the contractions concentrically, the probe was positioned using the abdominal aorta and the superior mesenteric artery as landmarks and the area was measured.

We induced caregiver hypnosis to the hypnoid-light hypnotic state at the end of tube feeding and maintained the state until 60 min after the end of feeding. The period of hand-holding was 60 min, beginning at the end of tube feeding. The method of hand-holding was not standardized, giving priority to the way each family acts in daily life. In addition, the participants were examined at intervals of 1–3 weeks, and they were alternately assigned to a group with relaxation or a group without relaxation, according to the entry order. The equipment used was the Toshiba Aplio300®, and the probe was 3 MHz convex.

Differences were assessed using Student’s paired *t*-test. The statistical analysis was performed using SigmaStat3.5, and the level of statistical significance was set at *P* < 0.05.

#### Evaluation of gastric motility result feedback

Feedback on the gastric motility results was provided for the family by giving a preview of the recorded ultrasonography and an explanation of the numerical values. We described the objective findings, but we did not explain the any assessment or suggestion. The feedback was provided within a week of the test day. Before and after receiving the feedback, three variables were assessed via self-administered questionnaires: guilt for self-care, willingness to adopt self-care and caregiver burden. Evaluation before receiving feedback was performed on the test day, and evaluation after feedback was performed two weeks after the test day.

Differences were tested using the Wilcoxon signed-rank sum test. Statistical analysis was performed using SigmaStat3.5, and the level of statistical significance was set at *P* < 0.05.

##### Experiment1

To assess willingness to adopt self-care, we asked “Would you like to adopt self-care?” and used a Numerical Rating Scale (NRS: an assessment method that converts the extent of symptoms into numerals; an 11-point scale ranging from 0 indicating no willingness to 10 indicating the highest willingness) [13]. For assessment of guilt about adopting self-care, we asked “Do you feel guilty about self-care?” again using the NRS (0 indicating no sense of guilt and 10 indicating extreme feelings of guilt).

##### Experiment2

The Japanese short version of the Zarit Burden Interview (J-ZBI_8: two-factor structure of personal strain-the burden caused by the care itself-and role strain-the burden caused by the impact of care on the caregiver’s life. Scored from 0 to 32 points: (Personal strain: 0–20 points, Role strain: 0–12 points) was used to assess caregiver burden [14, 15]. The reliability and validity of the J-ZBI_8 have been verified, and this interview schedule is widely used in clinical practice.

## Results

### Effects of hand-holding on gastric motility with or without hypnosis

**Fig. 2:**
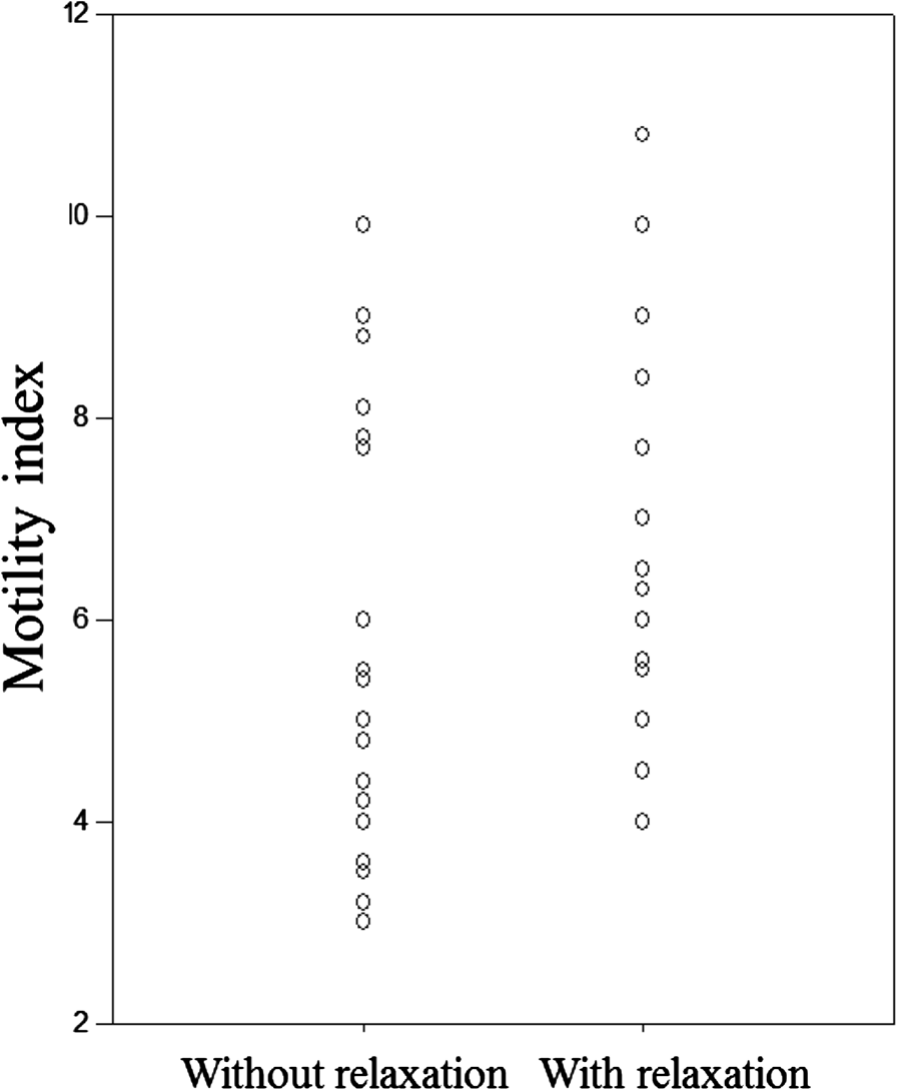
Motility index was 5.7 (SD = 2.0) without relaxation and 6.9 (SD = 2.3) with relaxation (*P* = 0.037)

**Fig 3:**
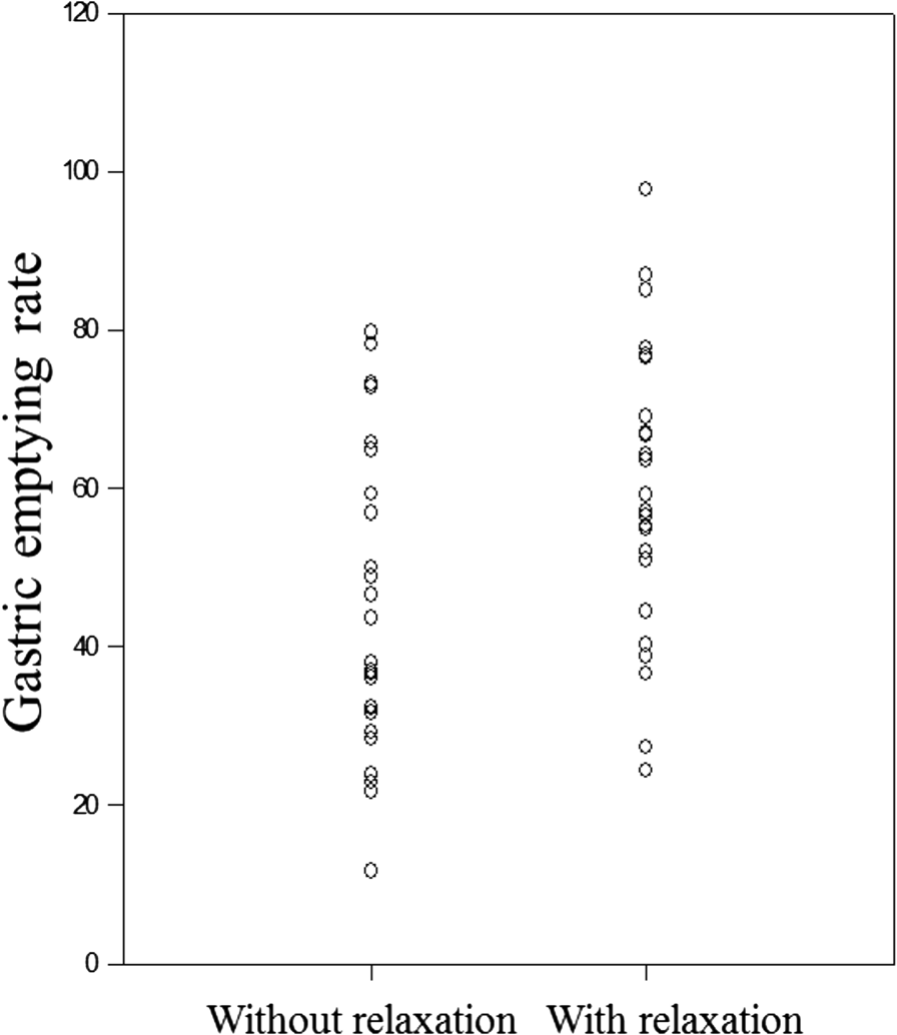
Gastric emptying rate was 44.9 % (SD = 19.0) without relaxation and 58.7 % (SD = 18.3) with relaxation (*P* = 0.010)

MI was 5.7 (SD = 2.0) without caregiver relaxation and 6.9 (SD = 2.3) with relaxation, and there was a significant difference between the two groups (*P* = 0.037) (Fig. 2). GER was 44.9 % (SD = 19.0) without relaxation and 58.7 % (SD = 18.3) with relaxation during the 60-min period after the end of tube feeding, and a significant difference was observed between the two groups (*P* = 0.010) (Fig. 3). Holding the patient’s hand while the family caregiver was experiencing relaxation significantly accelerated both the MI and the GER of the patients.

### Effects of gastric motility result feedback (Table 1)

**Table 1:** Evaluation of gastric motility result feedback

		Before feedback	After feedback	*p* value
NRS	Willingness to adopt self-care	3.3 (SD = 2.4)	6.4 (SD = 2.8)	*p* < 0.001
	Guilt for self-care	7.0 (SD = 2.6)	4.9 (SD = 2.9)	*p* < 0.001
J-ZBI_8	Total	14.3 (SD = 6.3)	10.8 (SD = 5.5)	*p* < 0.001
	Personal strain	8.3 (SD = 4.4)	7.5 (SD = 3.8)	*p* = 0.039
	Role strain	6.0 (SD = 2.8)	3.3 (SD = 2.5)	*p* < 0.001

Table 1: After receiving feedback on the gastric motility result, the caregivers’ willingness to adopt self-care increased and his or her sense of guilt decreased, which consequently reduced the caregiver burden

#### Experiment1

The NRS scores for willingness to adopt self-care were significantly higher after receiving feedback. The NRS scores for guilt to adopt self-care were significantly lower after receiving feedback. This suggests that the caregiver’s willingness to perform self-care was increased and his/her guilt was decreased by the feedback.

#### Experiment2

The J-ZBI_8 scores for caregiver burden were significantly lower after receiving feedback, suggesting that the caregiver burden was decreased. Both the personal strain and role strain subscales declined.

## Discussion

To our knowledge, this is the first report to study and evaluate an intervention that increases the willingness to adopt self-care, such as relaxation.

The first important finding in this study is that holding the patient’s hand while the family caregiver was receiving relaxation significantly accelerated two types of gastric motility function in the patients. Thus, this study has revealed information about the physiological changes caused by hand-holding by a family member in a state of relaxation. We consider that these changes may be a result of reflection on the autonomic nervous activity caused by the physical effect of hand-holding. In terms of the mechanism of action for this autonomic nervous system change, a deliberately added touch stimulus by hand-holding was passed from the receptors in the skin (the receptors responding to heat and cold, touch and pressure) to the hypothalamus, and reached the internal organs such as the digestive tract via the autonomic nervous center. Additionally, it is likely that there was a psychological effect. However, because the study participants were patients with a decreased level of consciousness, the psychological influence on the autonomic nervous activity can be considered minimal. In this case, it is thought that hand-holding affects the cerebral and limbic system via visual perception and receptors in the skin and is related to emotional aspects and higher brain functions. One report has suggested that the diastolic function of the proximal stomach was decreased when a sense of anxiety was experimentally induced, which influenced the limbic system and passed the stimulus from the hypothalamus through the autonomic nerve center [16].

Regarding the mechanism of action related to the autonomic nervous system in this study, relaxation affected the temperature and pressure senses of the caregiver, and similar impacts affected the patient’s skin by hand-holding. It has been reported that relaxation induced by self-hypnosis autogenic training inhibits efferent sympathetic nerve activity. The report showed that efferent sympathetic nerve activity inhibition increases the wave height and peripheral skin temperature measured by fingertip photoelectric plethysmograph, decreases the surface electromyography level, and causes skeletal muscle relaxation [17]. In this study, we could not prove this consideration because peripheral skin temperature was easily affected by external factors and skeletal muscle relaxation was not evaluated because of the hand-holding condition. There might be another influence associated with tactile contact, such as sweating. However, evaluating the influence of sweating is difficult, because sweating is reduced by decreased electrodermal activity resulting from the inhibition of efferent sympathetic nerve activity, while thermal sweating is increased by physical stimulus through hand-holding.

The second important finding in this study is that the caregiver’s willingness to adopt self-care was increased as a result of receiving feedback. The average NRS score for the willingness to adopt self-care before receiving feedback was as low as 3.3 points, whereas the score after receiving feedback was 6.4 points. It is likely that the caregiver’s significantly increased willingness was associated with his/her decreased guilt about adopting self-care after receiving feedback, suggesting that the family’s self-care could accelerate the autonomic nervous activity of the patient. It was reported that psychological needs, such as overcoming a sense of guilt in a family with a cancer patient, are often not met [5]. This study also showed that the average NRS score for guilt about performing self-care was as high as 7.0 points. It is known that the family’s sense of guilt is reduced when they have a meaningful relationship with the patient [6]. The present study also found that the average NRS score declined to 4.9 points after receiving feedback explaining that the family caregiver’s self-care was an intervention aimed at improving the situation for the patient.

In this study, the J-ZBI_8 for the evaluation of caregiver burden was as high as 14.3 points, indicating that caregiver burden was large. In terms of caregiver burden, Arai et al. reported in a study on the primary caregivers of the elderly who live at home and require long-term care that the J-ZBI_8 score was 9.3 points for caregivers troubled by care and 3.5 points for caregivers not troubled by care [15]. After receiving the feedback, the J-ZBI_8 score decreased significantly, reaching 10.8 points, which means that the caregiver burden was reduced. Considering the fact that the score on the role strain subscale declined remarkably, the caregiver’s increased willingness to adopt self-care could have a great effect on reducing the caregiver burden. Additionally, the reasons for the absolute value remaining as high as 10.8 points after receiving feedback might be the influence of the personal strain subscale and the early evaluation, which was performed just 2 weeks after the feedback was provided.

### Limitations

The present study has several limitations. First, we could not evaluate proximal gastric accommodation, which is an important factor of the gastric motility function in gastrostomy tube feeding. Reduced proximal gastric accommodation allows a larger amount of food to reach in the antrum of the stomach at once, which causes gastric emptying suppression because of the decreased antral motility and increased duodenal outflow [18]. Thus, proximal gastric accommodation is intricately associated with MI and GER. Second, there could be observer bias in the evaluation of gastric motility, but it is likely difficult to remove the bias because of the characteristics of hypnosis. Third, the manner of hand-holding was not standardized, giving priority to the way each family acts in daily life. We could not verify that differences in ways of hand-holding did not affect the results. However, considering the crossover study design, we do not consider it likely that these differences affected the results. Fourth, we did not verify that the changes in this study were the result of only reflection on the autonomic nervous activity (e.g., the immune system). Furthermore, we did not describe the autonomic nervous activity more specifically (i.e., parasympathetic, sympathetic or both systems), because the gastric motility test using ultrasonography cannot evaluated the parasympathetic and sympathetic systems separately. We feel that a balanced state of the parasympathetic and sympathetic systems enhances autonomic nerve activity. Fifth, because we did not objectively evaluate the hypnosis (e.g., through electroencephalogram), we could not verify that family caregivers were really relaxed by hypnosis. Furthermore, we could not verify that hypnosis induced only relaxation of the family caregivers. Hypnosis might affect the family caregiver’s hand-holding action itself. Hypnosis might affect other behaviors and the autonomic nervous activity of family caregivers. Finally, we did not sufficiently evaluate the reasons for increased willingness to adopt self-care. In addition to the reduced sense of guilt following feedback, relaxation might be increased by experiential awareness, which could be another reason for this increased willingness. However, we could not assess the reasons because we did not conduct a qualitative study with semi-structured interviews.

## Conclusions

Hand-holding by the family caregiver while he or she was experiencing relaxation enhanced the patient’s gastric motility function. By providing feedback on these results, the caregiver’s willingness to adopt self-care was increased and his or her sense of guilt regarding this self-care was reduced. This study suggests that a caregivers’ relaxation increases the patients’ autonomic nervous activity and that feedback on the positive results increases the caregivers’ willingness to engage in self-care, which consequently reduces the caregiver burden.

## Abbreviations

GER: gastric emptying rate
J-ZBI_8: Japanese short version of the Zarit Burden Interview
MI: motility index
NRS: numerical rating scale
